# A Mysterious Recurrent Urticarial Rash and a Life-Altering Metal

**DOI:** 10.7759/cureus.44469

**Published:** 2023-08-31

**Authors:** Adam Friedman, Olga Schweiker-Kahn, Satyajeet Roy

**Affiliations:** 1 Medicine, Cooper Medical School of Rowan University, Camden, USA; 2 Internal Medicine, Cooper University Hospital, Camden, USA; 3 Internal Medicine, Cooper Medical School of Rowan University, Camden, USA

**Keywords:** carotid stent allergy, nitinol, urticaria, nickel hypersensitivity, allergic contact dermatitis (acd)

## Abstract

Allergic contact dermatitis is a common manifestation in individuals who have a contact hypersensitivity to nickel. Direct exposure to nickel triggers an adaptive immune response that mediates a localized inflammatory reaction of the skin, typically resulting in an erythematous and pruritic rash at the site of contact. We present a distinct case in which nickel was systemically introduced via a carotid stent into an individual with an unidentified allergy to nickel. This case emphasizes the potentially life-threatening risks associated with implantable hardware containing metals, such as nickel. Moreover, this case highlights the potential benefit that screening for metal allergies may have before surgically implanting permanent metal-based devices.

## Introduction

Allergic contact dermatitis (ACD) is a form of type IV hypersensitivity reaction, specifically subtypes IVa and IVc, that occurs in response to an environmental antigen or allergen [1]. The pathophysiology of ACD begins when exogenous allergens make contact with and penetrate the epidermis and dermis, which induces an immune response mediated by sensitized CD4+ Th1-cells, CD8+ T-cells, and various cytokines [2]. The overall result is localized inflammation with signs of erythema, pruritus, edema, warmth, and pain at the site of exposure. If left untreated, lesions can progress to a chronic phase consisting of hyperkeratosis, dryness, fissuring, and lichenification [3].

ACD has a prevalence of about 20.1% in the general population, with the most commonly identified metal allergen affecting individuals being nickel [2,4]. Nickel is frequently incorporated into everyday items, such as currency, jewelry, and kitchen tools [5]. Nickel is also gaining utility in implantable medical devices, especially in the fields of orthopedic and cardiovascular surgery [6,7]. This permanent exposure to nickel raises concern for potentially fatal and systemic complications in patients with contact hypersensitivities to this metal. We present a rare case of systemically reactivated ACD in a patient with an undiscovered nickel allergy after the placement of a nickel-based carotid artery stent.

This case was previously presented as a meeting abstract and poster at the American College of Physicians - New Jersey Chapter, 2023 Annual Scientific Meeting, on March 10, 2023; the American College of Physicians, 2023 Internal Medicine Meeting, on April 29, 2023; and the 10th Annual Camden Scholars Forum on May 3, 2023.

## Case presentation

A 34-year-old female with a history of tobacco use, peripheral artery disease, and transient ischemic attack presented to the office with an intermittent pruritic rash on both of her hands for one week. She denied exposure to new outdoor environments, new food items, or ingredients, pets, soap, detergents, jewelry, etc. She had tried topical antihistamines, emollients, and steroids without significant relief. Two weeks prior to the onset of her symptoms, she had a left carotid artery stent placed after CT angiography revealed moderate stenosis, and she was started on apixaban and carvedilol. Her vital signs were within normal limits. Physical examination revealed erythematous urticarial patches on her palms and wrists. The rest of her physical examination was normal. A clinical diagnosis of urticarial dermatitis was made, and she was treated with methylprednisolone and hydroxyzine for symptomatic treatment.

**Figure 1:**
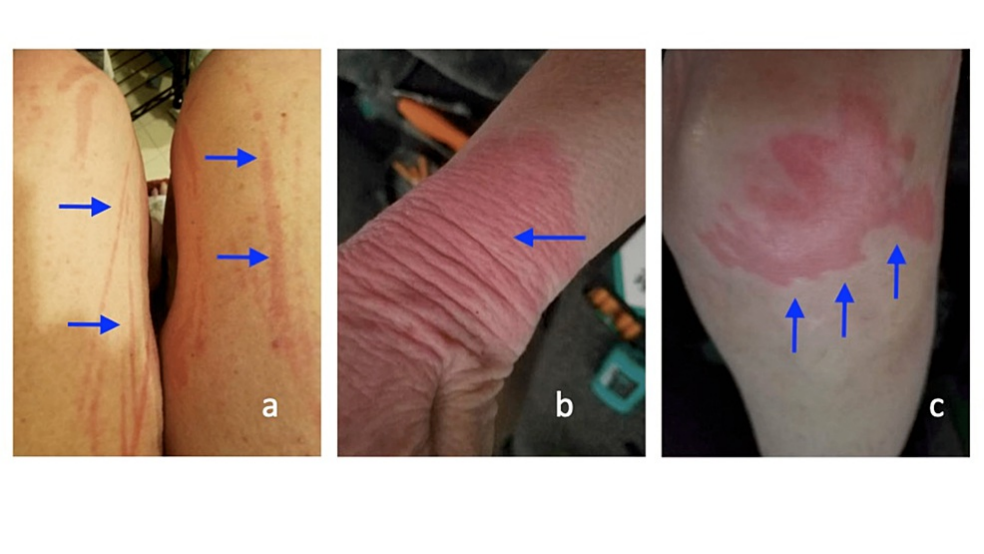
Images of the urticarial rash a) on the bilateral thighs with excoriations, b) on the right wrist with signs of dryness and lichenification, and c) on the anterior left knee with well-defined borders of erythema Provided with permission by the patient.

The patient’s symptoms persisted despite adherence to her initial treatment regimen. Differential diagnosis was expanded to consider either hypersensitivity or generalized adverse effects of her newly started medications. Carvedilol dosage was tapered and discontinued, with blood pressure remaining stable and no change in her dermatologic symptoms. Rivaroxaban was chosen as an alternative to apixaban, but the patient did not appreciate any difference. In a subsequent follow-up visit three months after the initial onset of symptoms, the patient presented with a similar but more diffuse rash that had spread to her back, abdomen, and lower extremities (Figure 1 a-c). She was also found to be hypotensive with a blood pressure of 89/62 mmHg. Given the concern for anaphylaxis, she was immediately transferred to the Emergency Department. Complete blood count, basic metabolic profile, electrocardiography, and coagulation studies were all unremarkable. The patient was admitted for a suspected anaphylactic reaction secondary to an unknown source, and she was treated with intravenous fluids and steroids. Once the patient was stabilized, further diagnostic workup was indicated to detect any previously undiscovered allergens. Extensive skin prick testing and patch testing concluded that she had a contact allergy to nickel. It was then confirmed that her left carotid stent was made of nitinol, a nickel-titanium alloy. Her left carotid artery stent was removed and replaced with a non-nickel-containing stent. At her two-week postoperative follow-up visit, the patient was elated to report that her generalized urticarial rash had completely resolved.

## Discussion

The presented case of an individual with an unidentified nickel allergy, who became chronically exposed to the allergen from her nickel-based carotid stent, is a unique and rare occurrence. Patients allergic to nickel can present with clinical manifestations of mixed-type hypersensitivity reactions, as well as systemic symptoms when ingested or introduced into the bloodstream [8]. Therefore, differential diagnosis should include more life-threatening allergic reactions, and anaphylaxis should be ruled out immediately upon presentation. In addition to contact allergens and fungal infections, systemic inflammatory diseases such as subacute cutaneous lupus erythematosus, psoriasis, and dermatomyositis should be considered due to their similar clinical presentations [9].

Management of typical contact allergy to nickel primarily consists of avoiding exposure to nickel-based compounds [2]. Initial strategies for managing ACD include moisturizers and emollients; however, if these are ineffective, then first-line pharmacotherapy is high-potency topical or oral corticosteroids, depending on the extent of cutaneous involvement [3,10]. In patients who recently started new prescription medications, concern for possible adverse effects should also be given. In the case of our patient, carvedilol was discontinued first due to its reported adverse effect of rash and hypersensitivity impacting 1-3% of those prescribed [11]. Apixaban was subsequently discontinued since adverse allergic skin reactions were reported to affect less than 1% of the prescribed population [12]. Given the lack of therapeutic benefit seen after initial treatment and discontinuation of newly initiated medications, extensive diagnostic patch testing was indicated to aid in detecting the causative agent [13].

## Conclusions

In conclusion, ACD is a common skin hypersensitivity that is frequently managed in the outpatient setting. Our case highlights a rather atypical presentation of ACD in which the manifestation of a systemic urticarial rash and anaphylaxis was secondary to a nickel-based nitinol carotid stent. This case of ACD required a high index of suspicion and prompt management by removal of the offending nickel-based agent, ultimately leading to a favorable outcome for our patient. The overall prognosis for nickel-induced contact allergy is excellent, and when considering our patient, she has achieved complete resolution of symptoms after the removal of the causative allergen.
